# Crowding Deficits in the Visual Periphery of Schizophrenia Patients

**DOI:** 10.1371/journal.pone.0045884

**Published:** 2012-09-26

**Authors:** Rainer Kraehenmann, Franz X. Vollenweider, Erich Seifritz, Michael Kometer

**Affiliations:** 1 Neuropsychopharmacology and Brain Imaging & Heffter Research Center, Clinic of Affective Disorders and General Psychiatry, Psychiatric University Hospital, University of Zurich, Zurich, Switzerland; 2 Clinic of Affective Disorders and General Psychiatry, Psychiatric University Hospital, University of Zurich, Zurich, Switzerland; Ecole Polytechnique Federale de Lausanne, Switzerland

## Abstract

Accumulating evidence suggests that basic visual information processing is impaired in schizophrenia. However, deficits in peripheral vision remain largely unexplored. Here we hypothesized that sensory processing of information in the visual periphery would be impaired in schizophrenia patients and, as a result, crowding – the breakdown in target recognition that occurs in cluttered visual environments – would be stronger. Therefore, we assessed visual crowding in the peripheral vision of schizophrenia patients and healthy controls. Subjects were asked to identify a target letter that was surrounded by distracter letters of similar appearance. Targets and distracters were displayed at 8° and 10° of visual angle from the fixation point (eccentricity), and target-distracter spacing was 2°, 3°, 4°, 5°, 6°, 7° or 8° of visual angle. Eccentricity and target-distracter spacing were randomly varied. Accuracy was defined as the proportion of correctly identified targets. Critical spacing was defined as the spacing at which target identification accuracy began to deteriorate, and was assessed at viewing eccentricities of 8° and 10°. Schizophrenia patients were less accurate and showed a larger critical spacing than healthy individuals. These results indicate that crowding is stronger and sensory processing of information in the visual periphery is impaired in schizophrenia. This is in line with previous reports of preferential magnocellular dysfunction in schizophrenia. Thus, deficits in peripheral vision may account for perceptual alterations and contribute to cognitive dysfunction in schizophrenia.

## Introduction

Deficits in basic visual information processing are a key impairment in schizophrenia. They are related to higher-order neurocognitive dysfunctions and functional outcomes [1]–[3] and are evident at incipient stages of schizophrenia before any psychotic symptoms occur [4]. However, the conditions under which dysfunctional visual processing occurs are unclear [5], [6]. Under natural viewing conditions, information from the visual periphery is essential for object and scene gist recognition, as well as for guiding eye movements to context-relevant locations [7], [8]. Previous studies on visual information processing in schizophrenia have largely neglected peripheral vision, and information regarding the presence, extent and nature of peripheral visual dysfunction in schizophrenia is incomplete and inconsistent [9]–[15]. For example, although Miller et al. [14] found no difference between central and peripheral visual processing in schizophrenia patients, Elahipanah et al. [10] found disproportionately large deficits when target stimuli were located peripherally, and Granholm et al. [11] identified peripheral deficits in schizophrenia patients that were most prominent when object density in the visual field was high. It therefore appears as though peripheral vision may be impaired in schizophrenia.

Crowding is a breakdown in object perception whereby one's ability to recognize a peripheral target is severely impaired by the presence of flanking objects [16]. Crowding in peripheral vision reduces the ability to recognize objects because they are too close together, and leads to a phenomenon whereby a single object in the periphery (“target”) becomes indistinct from nearby objects (“distracters”). As such, it is object spacing (target-distracter distance), and not object size (spatial resolution), that critically limits target-distracter discrimination in the periphery [16], [17]. Crowding is closely related to input processing in low-level visual cortices, where target and distracter signals are “compulsorily pooled” [18], [19]. Although this has the advantage of information compression, it comes at the cost of target-distracter discriminability [19]–[22]. In healthy individuals, crowding occurs when spacing falls below a critical value (critical spacing). This critical value of spacing increases as the visual angle between the fixation point and target (eccentricity) increases, and the approximately linear relationship between the two is been termed “Bouma's rule” [21].

It is suggested that peripheral visual stimuli are preferentially processed via the magnocellular pathway [23]–[26], which is disrupted in schizophrenia [27]–[29]. This is consistent with reports of peripheral vision deficits in schizophrenia patients [9]–[11], [15]. Therefore, we hypothesized that sensory processing of information from the visual periphery would be impaired in schizophrenia patients and, as a result, crowding would be stronger than in healthy individuals. To test this hypothesis we studied crowding in the visual periphery of both schizophrenic patients and healthy controls. We expected that accuracy in the crowding task would be lower, and the critical spacing larger, in schizophrenia patients than in healthy individuals.

## Materials and Methods

### Ethics statement

This study was approved by the Ethics Committee of the Canton of Zurich and was carried out in accordance with The Code of Ethics of the World Medical Association (Declaration of Helsinki, 2008 version). A clinician who was experienced in the evaluation of mental illness assessed by a direct examination of participants, their understanding of all the procedures and capacity to consent [30]. The participants were included in the study only if they had the full capacity to consent.

### Subjects

Twenty patients meeting the DSM-IV criteria for schizophrenia and 20 healthy subjects participated in the study. Schizophrenia patients comprised inpatients (n = 5) and outpatients (n = 15). Schizophrenia patients were recruited from the Psychiatric University Hospital Zurich and healthy controls were recruited by advertisement from the University of Zurich and the Zurich urban area. Diagnoses were obtained using the Mini International Neuropsychiatric Interview (MINI) [31] and available clinical information. Controls with a history of DSM-IV Axis I psychiatric disorder or substance dependence within the last year, as assessed by the MINI, were excluded. Patients and controls were excluded if they had a history of neurological or ophthalmologic disorders. All subjects were between 19 and 54 years old and had a corrected visual acuity of at least 0.6 according to the Freiburg Visual Acuity Test [32]. Groups were matched for age (t(38) = −1.51, p = 0.139), gender (Fisher's exact test, odds ratio  = 0.63, 95% confidence interval  = [0.12, 2.96], p = 0.731), IQ (t(38) = 1.76, p = 0.087) and visual acuity (t(37) = 0.48, p = 0.631). All patients were receiving antipsychotic medication at the time of testing. Chlorpromazine equivalents were calculated using conversion factors described elsewhere [33], [34]. The chlorpromazine equivalent dose of paliperidone is not adequately defined in the literature; therefore, the chlorpromazine equivalent dose was calculated from the defined daily dose set out in the WHO Collaborating Center for Drug Statistics Methodology Index 2011 (http://www.whocc.no/atc_ddd_index). A certified Positive and Negative Syndrome Scale (PANSS) rater (RK; The PANSS Institute LLC, NY) obtained PANSS ratings from patients. Demographics and clinical characteristics of subjects are presented in Table 1.

**Table 1:** Demographic and clinical characteristics of schizophrenia patients and healthy controls.

	Schizophrenia patients (n = 20)	Healthy controls (n = 20)
Age (y)	39.9 (9.66)	35.0 (11.2)
Gender (m/f)	13/7	15/5
MWT-B IQ	100.1 (15.3)	108.8 (16.0)
Visual acuity	1.31 (0.38)	1.36 (0.31)
Onset age (y)	22.6 (4.75)	
Illness duration (y)	16.8 (9.13)	
Lifetime admissions (n)	6.30 (5.29)	
Chlorpromazine daily equivalent (mg)	405 (377)	
Atypical antipsychotic medication(n)	20	
Typical antipsychotic medication(n)	4	
PANSS total score	80.8 (17.7)	
PANSS positive subscore	18.6 (6.12)	
PANSS negative subscore	20.5 (4.47)	
PANSS disorganization subscore	10.8 (2.97)	
RHS	8.70 (2.25)	

Values represent the mean (± standard deviation) unless otherwise indicated.

MWT-B IQ, Multiple Choice Vocabulary IQ [35]; PANSS, Positive and Negative Syndrome Scale [36], [37]; RHS, Revised Hallucination Scale, 6-item visual score [38].

### Apparatus

Subjects were tested in a dimly lit room (ambient illumination, 11 lux). Stimuli were presented using E-Prime software (Psychology Software Tools Inc., Pittsburgh, PA) and displayed on a liquid crystal display (Hewlett-Packard LP2065, resolution 1600×1200 pixels; Radeon HD4350 graphics card) placed 0.75 m in front of the subject. Head movement was constrained by a head-chin rest. During administration of the examination, the experimenter (RK) sat behind the computer screen and monitored eye movements and eye gaze direction in real time with an infrared eye camera. All participants reliably maintained central eye fixation. Furthermore, we reduced processing time by backward-masking and presented the stimuli in a randomized order on the left and the right sight of the screen to reduce the amount of eye movements towards the target stimuli.

### Visual stimuli

The visual stimuli comprised uppercase letters from the Roman alphabet. All letters were presented in a dark gray color on a white background (background illumination, 63 lux) and were of identical height and width (0.8° of visual angle). Four variables were manipulated: target, target-distracter spacing, the side of stimulus presentation, and the stimulus presentation eccentricity.

#### Target

The target letters were upright or 90° tilted uppercase “T”s, which appeared alone or flanked by uppercase distracter letters (“I” or “H”) above and below. The target and distracter letters were similar in appearance to increase the crowding effect [39].

#### Spacing

**Figure 1:**
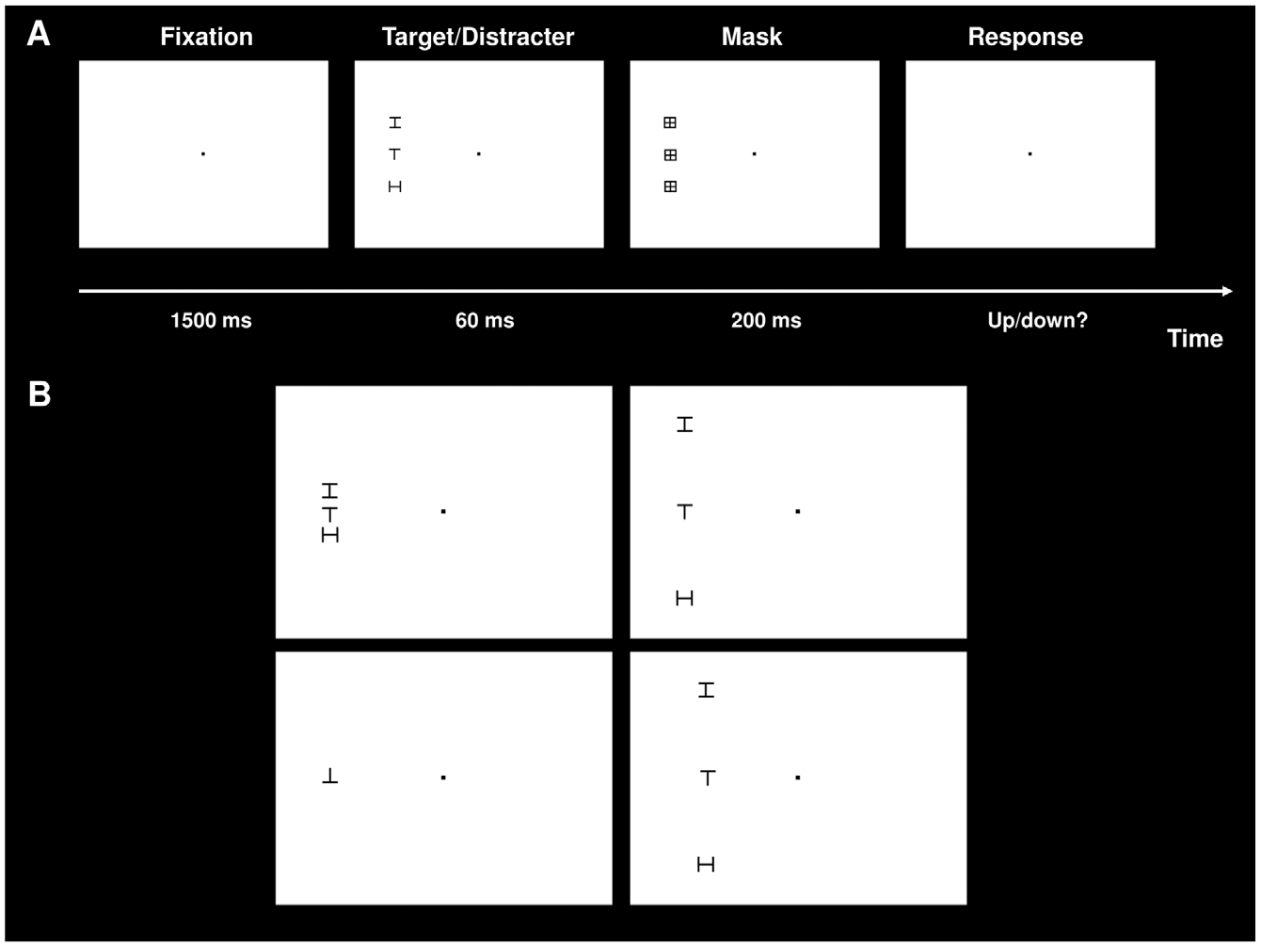
Schematic showing the crowding experiment and selected examples of stimuli used in the experiment. **Panel A:** A fixation point was first presented for 1500 ms, followed by a target/distracter array (4° spacing/10° eccentricity condition shown here) for 60 ms. Then, a mask appeared for 200 ms. Finally, a response screen displaying a fixation point was shown and the subjects were required to register whether they saw an upright or 90° tilted target “T” by pressing a key. **Panel B:** 2° spacing/10° eccentricity condition (top left); 8° spacing/10° eccentricity condition (top right); target-only/10° eccentricity condition, 90° tilted target “T” (bottom left); 8° spacing/8° eccentricity condition (bottom right).

Distracters appeared at one of seven equidistant locations on the vertical meridian of the targets. The target-distracter spacings (center-to-center) were 2°, 3°, 4°, 5°, 6°, 7° and 8° of visual angle (Figure 1).

#### Side

Targets and distracters appeared on either the left or right side of the central fixation point to ensure reliable central fixation, since selectively fixating at the left or right side of the central fixation point would be counterproductive for the subjects' accuracy performance.

#### Eccentricity

Targets and distracters appeared at either 8° or 10° of visual angle from the horizontal meridian.

### Experimental procedures

The crowding task has been previously described [40]. Figure 1 depicts a trial sequence in this crowding task. Subjects were instructed to fixate on a central point before stimulus onset, and to maintain central fixation during the trials. They were then asked to press the space key on the computer keyboard to initiate the trial. Initially, a fixation point (with a diameter of 0.2° of visual angle) appeared for 1500 ms. Next, target and distracter letters were displayed for 60 ms. This short interval between the stimuli interval precluded any eye movement [41]. Subsequently, masks appeared at the same target/distracter locations for 200 ms. Finally, a response screen displaying a fixation point was shown. At this time, the subjects were required to indicate whether they saw an upright or 90° tilted “T” by pressing the appropriate key on the computer keyboard. Following the response, a new trial was started. No feedback was given. The experiment consisted of six blocks of 64 trials each, giving a total of 384 trials. Within each block, all variables (target, spacing, side and eccentricity) were randomly intermixed. Spacing conditions appeared with equal probability (including a “target-only” condition where the target appeared without distracters). Between blocks, the subjects received a short break to avoid fatigue. Test blocks of 16 trials with visual feedback were performed before the experiment, and were repeated until the experimenter was sure that the subject understood the procedure.

### Calculation of critical spacing

Crowding occurs when target-distracter spacing falls below a critical value and recognition of the target letter is reduced. We computed the critical spacing value using a two-step algorithm. First, a logistic function (equation 1) was modeled to the data obtained from each individual subject, where *y* is the probability of correct target recognition and *x* is the corresponding target-distracter spacing:



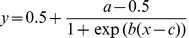
(1)


The constants a, b and c were used for standardization. Critical spacing was then computed from the fitted logistic function (equation 2) as the point of the curve where accuracy began to deteriorate [18], [40], [42]. We defined critical spacing according to Scolari et al. [40] and Yeshurun and Rashal [43], i.e., the point at which accuracy reached 90% of asymptotic performance:



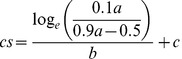
(2)


### Statistical analyses

All data were first tested for normality by means of a Shapiro-Wilk test. Accuracy was calculated as the proportion of correctly identified targets. Accuracy was then analyzed using a 2×7×2×6 mixed model analysis of variance (ANOVA) with group (schizophrenia, control) as a between-group factor, and spacing (2°, 3°, 4°, 5°, 6°, 7°, 8°), eccentricity (8°, 10°) and block (1, 2, 3, 4, 5, 6) as within-group factors. To control for group differences due to differential feature detection, attention, or task engagement effects, the effect of group on accuracy during the target-only condition was assessed using an unpaired t test for accuracy at each eccentricity (8°, 10°). Critical spacing was analyzed using a 2×2 mixed model ANOVA with group (schizophrenia, control) as a between-group factor and eccentricity (8°, 10°) as a within-group factor. When the ANOVA assumption of sphericity was violated, the Greenhouse-Geisser correction [44] was applied. Bonferroni-corrected post-hoc t tests were performed when ANOVA identified significant group main effects or interactions. Generalized eta squared (η_G_^2^) [45] or Cohen's d [46] were reported as measures of effect size. Pearson's product moment correlation was used to examine correlations between critical spacing and demographic/clinical variables. All significance levels were two-tailed with a preset α<0.05. If not stated otherwise, all values represent the mean (± standard deviation). The open source statistical software R, version 2.14.2 [47] was used for statistical analyses.

## Results

### Accuracy

**Figure 2:**
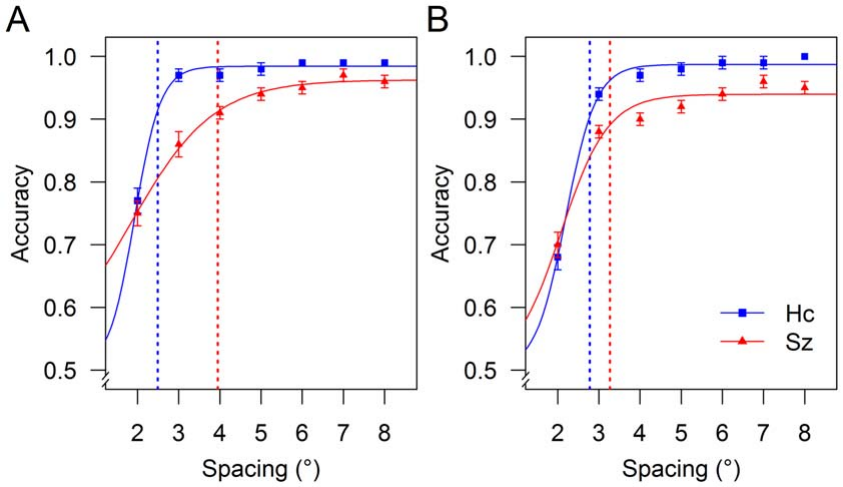
Accuracy and fitted logistic curves as a function of spacing at 8° eccentricity (panel A) and 10° eccentricity (panel B) in schizophrenia patients and healthy controls. Vertical dotted lines indicate critical spacing. Values represent the mean ± standard error of mean.

Figure 2 shows target identification accuracy as a function of spacing at 8° and 10° eccentricity in the patient and the control groups. Accuracy was significantly lower in the schizophrenia group than in the control group [F(1, 38) = 10.4, p = 0.003, η_G_^2^ = 0.09]. A significant main effect was found for spacing [F(2.17, 82.5) = 129, p<0.001, η_G_^2^ = 0.57] demonstrating increased accuracy with increased spacing. The main effect of target eccentricity was also significant [F(1, 38) = 8.38, p = 0.006, η_G_^2^ = 0.01] – accuracy decreased as target eccentricity increased. There was also a spacing × eccentricity interaction [F(3.78, 144) = 5.15, p<0.001, η_G_^2^ = 0.024]. At 8° eccentricity, accuracy was significantly lower in the schizophrenia group than in the control group at spacings of 3° [t(38) = 4.95, p<0.001, d = 1.56], 4° [t(38) = 3.13, p = 0.024, d = 0.99] and 6° [t(38) = 3.17, p = 0.021, d = 1.00]. At 10° eccentricity, accuracy was significantly lower in the schizophrenia group than in the control group at spacings of 4° [t(38) = 3.20, p = 0.019, d = 1.01], 5° [t(38) = 3.43, p = 0.010, d = 1.08] and 8° [t(38) = 3.84, p = 0.003, d = 1.22].

Accuracy during the target-only condition at 8° eccentricity was similar for both groups [t(38) = 1.64, p = 0.110, d = 0.52]. There was a tendency for schizophrenia patients to perform worse than controls at 10° eccentricity (accuracy of 0.96 (0.072) and 0.99 (0.023) respectively; t(38) = 1.97, p = 0.056, d = 0.62), but this was not significant. There were no differences of performance between groups due to vigilance decrements or training effects, as evidenced by a lack of a significant group × block, group × block × eccentricity, group × block × spacing and group × block × eccentricity × spacing interaction (all F≤1.45, p≥0.139, η_G_^2^≤0.010).

### Critical spacing

**Figure 3:**
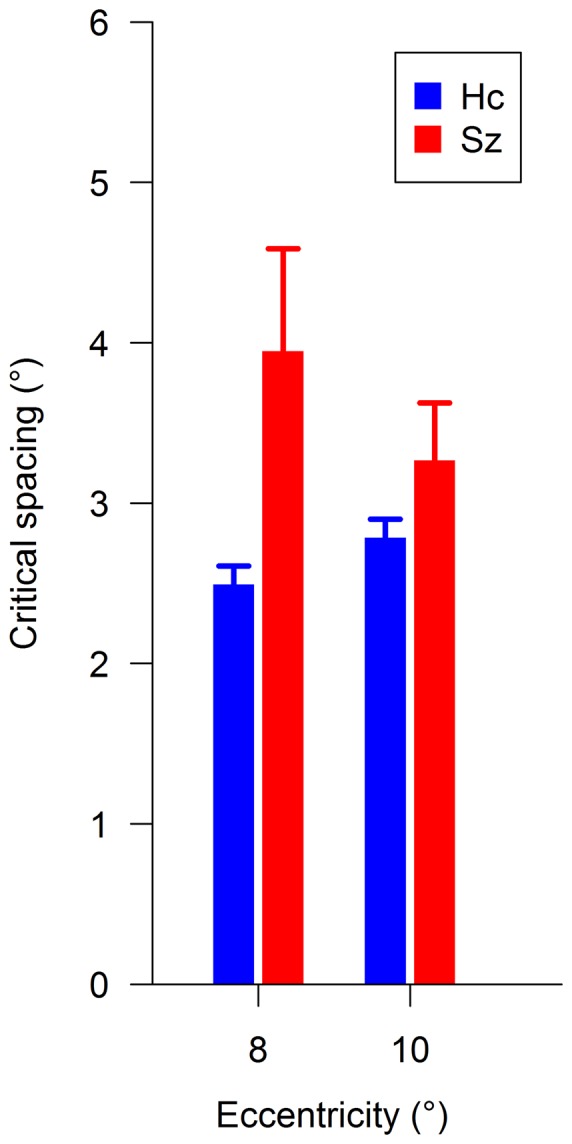
Mean critical spacing for schizophrenia patients (Sz) and healthy controls (Hc) by eccentricity. Critical spacing was larger in the schizophrenia group than in the control group. Error bars represent the standard error of the mean.

Logistic regression model fitting was very good for both patients (mean R^2^ = 0.97) and controls (mean R^2^ = 0.99). Critical spacing was significantly larger in the schizophrenia group than in the control group [F(1, 38) = 4.51, p = 0.040, η_G_^2^ = 0.08] (Figure 3). No main effect of eccentricity and no group × eccentricity interaction (all F≤3.15, p≥0.084, η_G_^2^≤0.022) were found.

### Relationship between critical spacing and demographic/clinical variables

There were no significant correlations between critical spacing and PANSS score (total, positive, negative, and disorganization), Revised Hallucination Scale (RHS) score, medication (chlorpromazine equivalents), visual acuity, intelligence quotient, age, or gender for the schizophrenia group (all Bonferroni-corrected p>0.100).

## Discussion

The results of this study provide the first evidence that visual crowding, a fundamental process in the visual periphery, is dysfunctional in schizophrenia patients. Visual crowding was greater in schizophrenia patients than in healthy controls, as evidenced by lower target identification accuracy and larger critical spacing. These results indicate that schizophrenia patients need more space between a target and distracters than healthy controls to correctly identify the target. This is consistent with our hypothesis of stronger crowding in schizophrenia, and indicates impaired sensory processing of information in the visual periphery.

### Magnocellular system

Converging lines of evidence indicate that information processing in the visual periphery is mediated by magnocellular neurons, whereas foveal processing is mediated by parvocellular neurons [26], [48]. Interestingly, Omtzigt et al. [49] compared the identification of parafoveally-presented flanked and unflanked target letters in healthy subjects, and found that the magnocellular system was specifically involved in the identification of flanked letters. This underpins the role of the magnocellular system in tasks where target and distracters are closely spaced, such as the crowding task in the present study. The finding that schizophrenia patients have deficits in the crowding task is consistent with previous studies showing robust magnocellular deficits in schizophrenia patients [27]–[29], [50]. N-methyl-D-aspartate (NMDA) receptor dysfunction may underlie these magnocellular deficits [28] and may therefore be a critical pathogenetic mechanism of crowding deficits in schizophrenia. It has been demonstrated [51]–[54] that, in schizophrenia, magnocellular dysfunction leads to increased intrinsic neural activity, which in turn elevates noise levels during signal processing in early visual cortex. According to signal detection theory, increased internal noise at the sensory level reduces target-distracter discriminability, which may in turn increase critical spacing [18], [55]. Therefore, the larger critical spacing in schizophrenic patients observed here might be a result of increased intrinsic noise due to magnocellular dysfunction. One might argue that increased noise at higher-level processing stages, including attention and decision making, might also reduce accuracy and therefore lead to a larger critical spacing. However, it is important to keep in mind that critical spacing is a relative measure of accuracy and is calculated as 90% accuracy relative to asymptotic performance. Therefore, although increased noise levels at higher-level processing stages may indeed reduce accuracy levels, the critical spacing effect will still be evident.

Interestingly, it has been shown that noisy sensory processing in subcortical areas may lead to secondary cortical processing impairments in schizophrenia [56]–[58]. Indeed, crowding deficits may themselves lead to downstream cognitive dysfunctions such as impaired perceptual decision making. Baldassi et al. [59] showed that intrinsic noise may account for perceptual decision errors under crowding conditions. Such perceptual decision errors are usually made with high confidence, and the underlying cortical activity in the sensory visual cortex strongly correlates with the subjective percept [60]. This may have implications for the understanding of bottom-up contributions to hallucination and delusion formation in schizophrenia, as several lines of evidence indicate that fixed, false beliefs may arise from erroneous sensory processing [54], [61]–[64]. Our finding of larger critical spacing in schizophrenia implies a smaller uncrowded window and a more corrupted visual field, compromising the quality of visual input to thalamic nuclei [61]. This, in turn, may damage coherent thalamocortical oscillations, which are critical to normal cognitive functioning. Moreover, deficits in synchronized neural oscillations have been related to disconnectivity between and within cortical areas and thus may underlie the fragmentation of mind and behavior in schizophrenia [65]. Indeed, patients report impressions such as “If I look at my watch, I see a watch, watchstrap, face, hands and so on, then I have got to put them together to get it into one piece” [66].

### Perceptual organization

In crowding, the perception of a peripherally viewed target is impaired by adjacent distracters, leading to a cluttered percept. As described in the introduction, crowding is usually considered to result from spatial pooling of information that yields the perception of a textural representation of the visual periphery. Although the neuronal correlates and computations that result in crowding are still undetermined, it is assumed that the underlying mechanisms comprise contour integration [67], feature binding [68], [69] and spatial attention [70]. Converging lines of evidence indicate that schizophrenia patients are impaired in their ability to organize low-level visual information into coherent patterns such as groups, contours, perceptual wholes and object representations [71]–[75]. For example, Silverstein et al. [74] used a psychophysically well-controlled contour integration paradigm and found that schizophrenia patients performed poorly if they had to detect a smooth contour among discrete but aligned elements embedded in a background of random distracters. In addition, Must et al. [71] reported that, in schizophrenia patients, the detection of an oriented target is less facilitated by the presence of collinear flankers than in healthy individuals and Dakin et al. [72] showed that suppression of visual context is weaker in schizophrenia patients than in healthy subjects. Such perceptual organization deficits have been related to abnormal lateral interactions of local processing units in early visual cortex [76].

It has been shown [69], [77] in healthy subjects that target-distracter similarity, or good continuation between target and distracters, leads to perceptual grouping and thus increases crowding, whereas target-distracter dissimilarity or “wiggle” of target and distracter elements alleviates crowding. Therefore, perceptual organization deficits in schizophrenia patients may be expected to result in weaker crowding. However, our finding of stronger crowding in schizophrenia is contrary to this expectation. There are several possible explanations for this inconsistency. First, previous studies using perceptual organization tasks in schizophrenia patients may have favored central over peripheral visual processing because stimuli were presented centrally rather than peripherally. Perceptual organization deficits observed in central vision might differ from those observed in peripheral vision, a view corroborated by May et al. [78], who showed that contour integration may be strongly impaired by crowding effects at extreme eccentricity. Second, Hess et al. [79] reported that contour linking due to long-range horizontal interactions is absent in the visual periphery. Therefore, it is conceivable that the neural mechanisms underlying perceptual organization may differ between the fovea and the visual periphery. Third, it has been shown [74], [80] that perceptual organization deficits strongly correlate with the disorganized syndrome of schizophrenia and that clinically stabilized outpatients may lack perceptual organization deficits. In fact, we found no significant correlations between crowding measures and the PANSS disorganization score, and 75% of the patients in our study were stabilized outpatients. Therefore, it seems plausible that perceptual organization deficits do not account for the crowding deficits that we observed.

### Spatial attention

Dysfunctional spatial attention might better explain increased crowding in schizophrenia patients. Several studies [70], [81], [82] have shown that visual crowding may result from limitations set by spatial attention, and accumulating evidence [83]–[85] indicates that spatial attention is impaired in schizophrenia. Moreover, deficits in spatial attention in peripheral vision may be related to deficits in the magnocellular system, also termed the “where” pathway, because this is the system that mediates the perception of spatial relationships in the visual periphery [23], [24], [86]. Increased crowding in schizophrenia would be in line with a more limited peripheral visual system due to dysfunctional spatial attention. The interaction between crowding and spatial attention may be better understood in light of the findings of Zhang et al. [87]. They showed that in order to improve localized visual discrimination, the primary visual cortex constructs a bottom-up saliency map of visual space, which then guides attentional shifts by reporting local attentional attraction. Saliency maps are important processing interfaces in crowding [88] and visual search [89]. Results from electrophysiological and neuroimaging studies [82], [90]–[93] also indicate that the interaction between spatial attention, magnocellular processing and crowding may be mediated by sensory visual cortical areas, which is in line with the evidence of impairments at the earliest stages of visual processing in schizophrenia patients [76].

### Visual search and span of apprehension

Our findings may help to explain the conflicting results reported by previous studies on visual search in schizophrenia [9]–[15]. There is increasing evidence that crowding critically modulates the performance of visual tasks requiring detection of a target amidst multiple distracters [41], [94]–[97]. Vlaskamp and Hooge [98] showed that crowding reduces target-distracter discriminability and slows visual search times by up to 76%. In addition, crowding has been closely associated with the “functional visual field” or “span of apprehension”, i.e., the radial area around the fixation point from which information can be extracted at a glimpse [99]. The boundary of this area is defined as the eccentricity beyond which crowding occurs. Target and distracters inside this boundary appear uncrowded; thus, it is termed the “uncrowded window” [16]. Our finding of larger critical spacing in schizophrenia patients indicates a smaller functional visual field, and this is supported by a number of studies that reported a smaller functional visual field in schizophrenic patients [9], [10], [100], [101].

### Developmental dyslexia

A smaller functional visual field as a result of increased crowding has also been reported in dyslexic subjects, where, due to the detrimental effect on letter discriminability, it is interpreted as an important constitutive factor for reading deficits [102], [103]. Although a direct link between schizophrenia and dyslexia remains to be established, substantial evidence [104], [105] indicates that a variety of characteristics, including visual processing deficits, visual anomalies of perception, mixed handedness and reading impairment, are common to both disorders and may be a consequence of a shared underlying pathogenetic mechanism. This shared mechanism may also underlie deficits in other perceptual domains, such as auditory processing [106] and multimodal integration [107]. Structural and functional brain abnormalities of cortical regions surrounding the temporoparietal junction have regularly been found in dyslexia and schizophrenia and may be candidate loci of visual dysfunction in these disorders, as they are closely linked to auditory processing, orienting of spatiotemporal attention, and reading acquisition [108]. However, converging lines of evidence [104], [105], [109], [110] indicate that it may be subcortical magnocellular dysfunction that underlies both schizophrenia and dyslexia, in particular with regard to visual processing and associated cognitive abnormalities. This is supported by the finding that, in both disorders, structural and functional lateralization is reduced, as evidenced by abnormal symmetry of the planum temporale and a high rate of mixed handedness [110], [111], and reduced lateralization [106] has been attributed to magnocellular dysfunction. Thus, magnocellular dysfunction may be an important pathophysiologic mechanism underlying visual processing deficits in both schizophrenia and developmental dyslexia.

### Face recognition

A similar mechanism may also contribute to face recognition deficits in schizophrenia, although current evidence to support this hypothesis is equivocal [28]. On the one hand, there is evidence that abnormal structural and functional deficits of the fusiform face area, a temporal cortical region relevant to processing of faces, may primarily mediate the well-documented face recognition deficits in schizophrenia [112]. On the other hand, it has been suggested that activity of the fusiform face area is preserved when processing faces [113] and that basic visual processing deficits related to magnocellular dysfunction, along with their amplified modulatory effect on the fusiform face area, might better account for previous findings [114], [115]. We therefore suggest that crowding dysfunctions may substantially contribute to face recognition deficits in schizophrenia. This hypothesis is supported by Shin et al. [116], who reported that schizophrenic patients exhibited extremely poor facial recognition when they had to discriminate faces with different spacing between facial features. Additionally, Martelli et al. [117] showed that, in healthy subjects, crowding occurs among facial features within a single face and thus may severely impair face recognition. However, whether or not the crowding of facial features affects face recognition in schizophrenic patients remains to be tested in future studies.

### Compensatory mechanisms

As discussed above, dysfunctional crowding in schizophrenia may be related to perceptual alterations and cognitive disturbances. However, in this study, we did not find any significant correlations between crowding measures and clinical symptoms, perhaps because our clinical sample consisted primarily of clinically stabilized outpatients. The absence of any correlation indicates that the observed crowding deficits were not related to symptoms, and thus may be considered permanent and stable. It is therefore conceivable that the patients' brain may have adapted to, at least in part, compensate for crowding deficits. A global compensatory mechanism was reported in brain-damaged patients with spatial neglect after they wore an optical prism and was attributed to a recalibration of internal spatial maps by fronto-parietal networks [118]. The poor quality of sensory data in schizophrenic patients may likewise necessitate increased top-down control to enable them to make sense of their visual world. On a neural level, top-down control of sensory perception is implemented by frontal and parietal cortical areas through modulation of visual cortex activity [119]. Indeed, compensation of low-level visual deficits through increased recruitment of higher-level cortical areas has consistently been reported in schizophrenic patients [120]–[123]. For example, Knebel et al. [120] used visual evoked potentials to show that, in parafoveal vision of schizophrenic patients, deficits of early visual processing are compensated for later in the visual hierarchy.

In addition to top-down control strategies, patients may also adapt under natural conditions their eye and/or head movement pattern to compensate for crowding deficits. Because they have a smaller uncrowded window, the amount of information they can extract at a glance is reduced. Consequently, they would need to increase the number of fixations to compensate for this deficit. However, evidence to support this prediction is inconclusive, perhaps because low- and high-level deficits in cortical processing may lead to different eye scanning abnormalities in schizophrenia. Abnormal smooth pursuit and antisaccades, for example, are well documented in schizophrenia and probably reflect deficits in prefrontal cortex, specifically in the frontal eye fields [124]. On the other hand, compensatory eye and/or head movement patterns due to a smaller functional visual field may also be plausible in schizophrenia. Olevitch et al. [125] registered spontaneous head movements of schizophrenic patients during a reading task and found that patients initiated head movements at a smaller visual angle than controls, and Roberts et al. [109] used a psychophysically well-controlled reading paradigm and found that the number of saccades was increased and the observed eye movement patterns were closely related to reduced sensitivity to parafoveal information in schizophrenia patients.

One may think that another strategy to compensate for a smaller functional visual field would be to increase the viewing distance. However, if fixation is maintained on a point in the scene while viewing distance is increased, target size and eccentricity both decrease in proportion to the spacing of target and distracters. Although this “zooming out” will broaden the focus of the scene, the stimulus input at the retina leaves the critical spacing unchanged [16]. Therefore, increasing viewing distance might not be a viable strategy for schizophrenic patients to compensate for a smaller functional visual field. However, as far we are aware, no studies to date have systematically examined visual performance of schizophrenic patients in relation to viewing distance. It would be informative to test this relationship in a future study.

### Limitations

All targets and distracters were masked with overlapping high-energy backward masks to minimize the processing time for the stimuli. This reduces eye movements towards target stimuli and therefore ensures peripheral processing. However, backward-masking deficits have been documented in schizophrenia [126], [127] and thus may have confounded the observed crowding effects. Although we cannot exclude this possibility entirely, we consider it unlikely for two reasons. First, all stimuli in the crowding task were equally masked across all conditions, which is contrary to the condition-specific deficits observed, and second, although target detection is differentially modulated by masking and crowding, feature detection is impaired in masking but spared in crowding [18]. Our results show that feature detection was equal in both groups, as evidenced by similar accuracy levels during the target-only condition. We therefore conclude that the crowding effects are specific and not confounded by masking effects.

The small sample size and the higher variance of critical spacing in the schizophrenia group compared with the control group means that this study was underpowered for detecting critical spacing deficits at both eccentricities. This might explain the lack of an eccentricity effect and a group by eccentricity interaction on critical spacing in the ANOVA's. In addition, to ensure that the length of the testing session was tolerable to the participants, we only tested visual crowding at two eccentricities. Therefore, we cannot make direct conclusions about the full extent of the visual periphery. It would be very interesting to examine critical spacing in schizophrenia patients across a broader range of eccentricities in future studies.

### Conclusions

This study provides evidence that processing in the visual periphery of schizophrenic patients is impaired. Most notably, we report for the first time that crowding, a critical and ubiquitous process of peripheral vision, is impaired in schizophrenia. Our findings indicate that it is important to consider object spacing in relation to eccentricity in future studies of visual processing in schizophrenia, and that studying crowding might help us better understand visuospatial deficits associated with this illness. In particular, our findings imply that crowding deficits in schizophrenia might underlie perceptual alterations and cognitive dysfunction. For future studies, it would be enlightening to examine the relationship between visual crowding and magnocellular-biased processing, as well as cognitive, emotional and social functioning in a large sample of schizophrenia patients, preferably using multi-sensory modalities.
